# Is the Secret of VDAC Isoforms in Their Gene Regulation? Characterization of Human *VDAC* Genes Expression Profile, Promoter Activity, and Transcriptional Regulators

**DOI:** 10.3390/ijms21197388

**Published:** 2020-10-07

**Authors:** Federica Zinghirino, Xena Giada Pappalardo, Angela Messina, Francesca Guarino, Vito De Pinto

**Affiliations:** 1 Department of Biomedical and Biotechnological Sciences, University of Catania, Via S. Sofia 64, 95123 Catania, Italy; federica.zinghirino@phd.unict.it (F.Z.); xena.pappalardo@phd.unict.it (X.G.P.); vdpbiofa@unict.it (V.D.P.); 2 Department of Biological, Geological and Environmental Sciences, Section of Molecular Biology, University of Catania, Viale A. Doria 6, 95125 Catania, Italy; angela.messina@unict.it; 3 National Institute for Biostructures and Biosystems, Section of Catania, 00136 Rome, Italy; 4 We.MitoBiotech S.R.L., c.so Italia 172, 95129 Catania, Italy

**Keywords:** VDAC isoforms, gene structure, expression profile, core promoter, transcription factor binding sites, mitochondrial function

## Abstract

VDACs (voltage-dependent anion-selective channels) are pore-forming proteins of the outer mitochondrial membrane, whose permeability is primarily due to VDACs’ presence. In higher eukaryotes, three isoforms are raised during the evolution: they have the same exon–intron organization, and the proteins show the same channel-forming activity. We provide a comprehensive analysis of the three human *VDAC* genes (*VDAC*1–3), their expression profiles, promoter activity, and potential transcriptional regulators. VDAC isoforms are broadly but also specifically expressed in various human tissues at different levels, with a predominance of VDAC1 and VDAC2 over VDAC3. However, an RNA-seq cap analysis gene expression (CAGE) approach revealed a higher level of transcription activation of *VDAC3* gene. We experimentally confirmed this information by reporter assay of *VDACs* promoter activity. Transcription factor binding sites (TFBSs) distribution in the promoters were investigated. The main regulators common to the three *VDAC* genes were identified as E2F-myc activator/cell cycle (E2FF), Nuclear respiratory factor 1 (NRF1), Krueppel-like transcription factors (KLFS), E-box binding factors (EBOX) transcription factor family members. All of them are involved in cell cycle and growth, proliferation, differentiation, apoptosis, and metabolism. More transcription factors specific for each *VDAC* gene isoform were identified, supporting the results in the literature, indicating a general role of VDAC1, as an actor of apoptosis for VDAC2, and the involvement in sex determination and development of VDAC3. For the first time, we propose a comparative analysis of human VDAC promoters to investigate their specific biological functions. Bioinformatics and experimental results confirm the essential role of the VDAC protein family in mitochondrial functionality. Moreover, insights about a specialized function and different regulation mechanisms arise for the three isoform gene.

## 1. Introduction

The VDAC (voltage-dependent anion-selective channel) is the prototype of the family of subcellular pores responsible for the permeability of the mitochondrial outer membrane [1]. This protein is a key control point for the passage of ions and metabolites and thus, guarantees cell energy production. During evolution, more isoforms were raised in the eukaryotic organisms [2]: they were characterized on the basis of their functional traits, revealing very close permeability features [3,4,5,6,7]. In addition, experiments aimed to study their expression patterns in different tissues indicated very subtle differences and a quite ubiquitous presence in the tested tissues [8].

In general, the distribution of VDAC isoforms is more or less ubiquitous in tissues but with a prevalence of VDAC1 isoform. Although the three *VDAC* genes codify proteins with apparently the same function, differences in their amino acid content, the mitochondrial outer membrane localization [9], the channel functionality and voltage dependence [6,7], and the contribution of the N-terminal portion to cell viability and survival [10], lead to the hypothesis of a more specialized role/function for each isoform in different biological contexts.

Results from various groups highlighted specific functions for each isoform, and it is considered a common notion that, for example, VDAC1 is a pro-apoptotic actor [11], while VDAC2 is an anti-apoptotic protein [12]. The role of VDAC3 was associated with sex tissue development and maintenance soon after its discovery [13,14]. A further step was the deletion of the single gene isoform, which was performed in transgenic mice. In VDACs knock-out mice, physiological defects are linked to altered structure and functionality of mitochondria [11,13]. Mitochondria lacking VDAC (*VDAC1*−/−) are characterized by increased size, irregular and compacted cristae, and altered oxygen consumption either in oxidative either in glycolytic muscle fibers [11]. Other alterations have been detected in specific tissue injuries or pathologies, such as Alzheimer’s disease [15,16]. Impaired oxidative phosphorylation was detected in muscle biopsies of child patients affected by encephalomyopathy. This altered functionality of mitochondria was associated with the absence of VDAC1. [17,18]. Embryonic stem cells *VDAC2*−/− knock-out were obtained and have been used to generate knock-out mice. However, the lack of VDAC2 determined embryo lethality [19]. A significant degree of growth retardation characterizes *VDAC1/3* −/− mice; and *VDAC3* −/− deficient male mice show the peculiarity of infertile, with a disassembled sperm tail, the flagellum essential for sperm motility [13]. More recently, the amino acid composition of the three mammalian VDAC isoforms was examined, showing interesting peculiarities [20]. The cysteine content of the three isoforms is different, and this was correlated to their oxidation and potential disulfide bond formation [21]. The three isoforms carry strikingly different amounts of cysteine whose different role has been hypothesized [20].

Similar to VDACs knock-out mice, defects in the brain, muscle, and germinal tissues function were observed in *Drosophila melanogaster* VDAC1 mutants [22]. In the yeast *Saccharomyces cerevisiae*, two porin genes were found, but only *POR1* coding VDAC1 is essential for life [23].

A peculiar expression of VDAC isoforms was observed in cells and tissues of the germinal lines of different organisms. While VDAC1 is mainly located in cells of reproductive organs necessary to support the development of gametes [24,25], VDAC2 and VDAC3 are expressed in a specific portion of sperm and oocyte, and genetic variants or the aberrant regulation of these genes are correlated with infertility [26,27].

Mechanisms of *VDAC* gene expression regulation have never been explored in a comparative and systematic way. Only one paper has been published, describing the organization and activity of mouse VDAC gene promoters [28]. A prediction of the regulatory regions of the three *VDAC* genes shows that the promoters lack the canonical TATA-box and are G+C-rich. The transcription start site (TSS) was identified, and the mouse putative *VDAC* promoters tested for their activity. In more recent years, few other reports have been published on *VDACs* promoter regulation for specific germinal lineage. The activity of *VDAC2* promoter in a mammal’s developing ovary triggered by GATA1 and Cellular and viral myb-like transcriptional regulators (MYBL2) transcription factors lead to autophagy inhibition confirming its relevant role in cell survival [29]. In human male abnormal hypermethylation of *VDAC2* promoter correlated with idiopathic asthenospermia while in complete unmethylation or mild hypermethylation, sperm motility improved confirming the role of VDAC2 expression in human spermatozoa [30]. The increased expression level of the VDAC1 transcript was also induced by a lncRNA through enhancement of H3K4me3 levels in its promoter, leading to apoptosis of placental trophoblast cells during early recurrent miscarriage [31]. The present work proposes, for the first time, a study of the genomic region located upstream the TSS generating the three VDACs mRNA. We collected the information on human *VDAC* genes structure and transcription from the main publicly resources. Once *VDAC* gene promoters were identified, we analyzed the sequences using bioinformatics software to identify transcription factor binding sites (TFBSs) distribution. We performed experimental tests using a molecular biology approach to confirm the insights obtained and to assess the transcriptional activity. We believe that understanding the molecular mechanisms triggering *VDAC* genes transcription in physiological and altered conditions might highlight the biological role of each isoform inside the cells and in different biological contexts.

## 2. Results

### 2.1. Structure of Human VDAC1, VDAC2, and VDAC3 Genes: Transcripts Variants and Promoters

The vast amount of large-scale genomic projects, high-throughput sequencing, and transcriptomic data, as well as the plentiful supply of promoter resources, assist in the comprehensive reconstruction of a transcriptional regulatory region. The intersection of this data enabled us to provide a framework of the main functional DNA elements for the identification of active biochemical regions, which are commonly understood as gene promoter sequences of each *VDAC* isoform, and to study the transcriptional control of the promoter structure via the analysis of TF-binding sites specificity and co-association patterns with other TFs.

The present work combines this evidence with information from gene expression profile datasets and with analysis of the promoter-specific activity of *VDACs*, which helps to investigate the co-expression relationship and the context-dependent regulation of *VDAC* genes.

**Figure 1:**
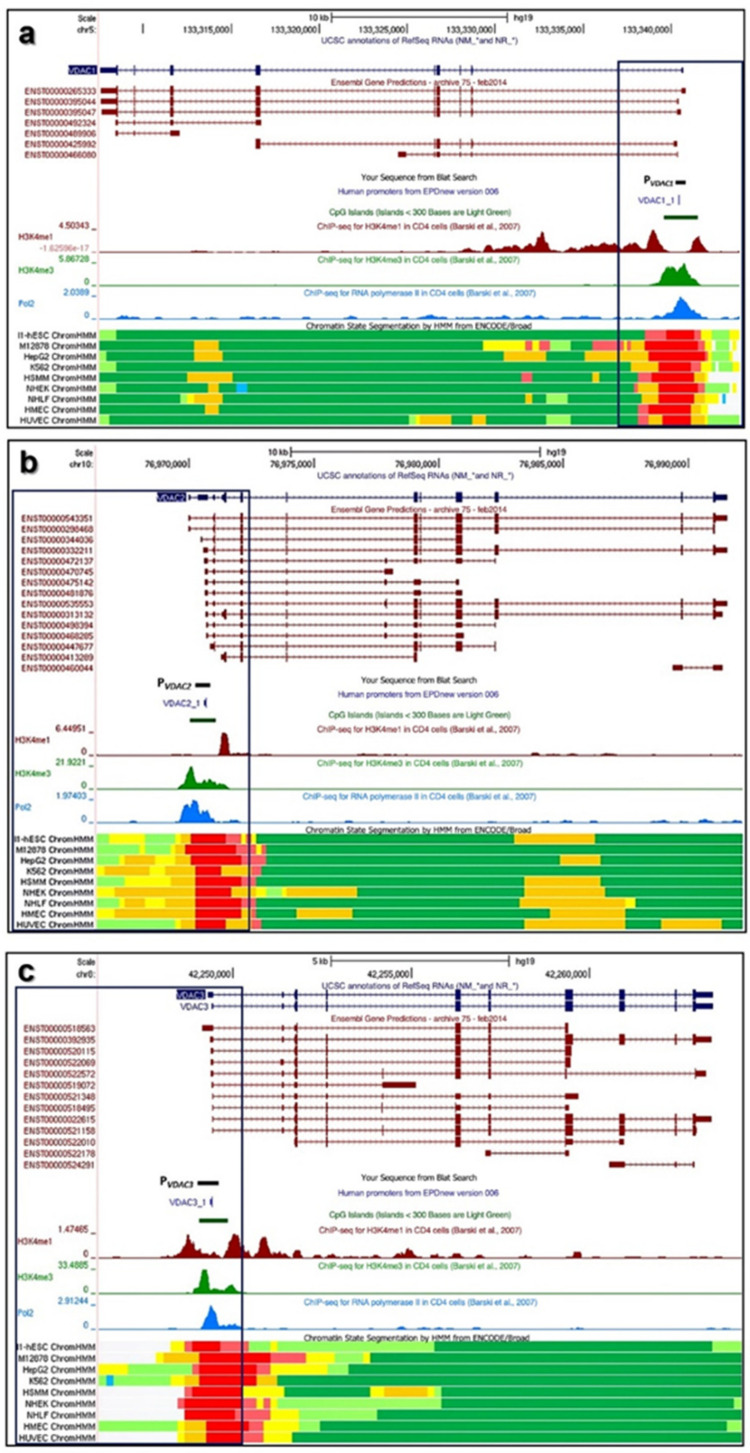
Human *VDAC* (voltage-dependent anion-selective channels) isoforms gene structures and functions. Overview of the gene structure of human *VDAC* isoforms and their most relevant functional and structural sites. (**a**) *hVDAC1* gene location on Chr5:133,340,230-133,340,830 (GRCh37/hg19) from UCSC Genome Browser; (**b**) *hVDAC2* gene location on Chr10:76,970,184-76,970,784; hg19; (**c**) *hVDAC3* gene location on Chr8:42,248,998-42,249,598; hg19. In each panel, a black box encloses the 600 bp promoter sequence indicated as P*_VDAC1_*, P*_VDAC2_*, and P*_VDAC3_*, respectively, and aligned with the annotated sequence from EPDnew (v.006). This allows the profile of transcriptional activity of the gene promoter region to be highlighted by CpG island identification, levels of enrichment of the H3K4me1 and H3K4me3 histone marks, and RNA Pol2 and Chromatin State Segmentation ChIP-seq data. Functional elements of Chromatin state segmentation by HMM of nine different cell lines are identified using different colors as follows: bright red: active promoter; light red: week promoter; orange: strong enhancer; yellow: weak/poised enhancer; blue: insulator; dark green: transcriptional transition/elongation; light green: transcriptional transcribed.

Our approach narrowed down the analysis of the promoter region of each *VDAC* isoform to defined 600bp segment for P*_VDAC1_* (chr5:133,340,230-133,340,830; hg19), P*_VDAC2_* (chr10:76,970,184-76,970,784; hg19), and P*_VDAC3_* (chr8:42,248,998-42,249,598; hg19), whose range is from −400 bp to +200 bp relative to TSS of annotated promoter sequences in EPD new (v.006). The boxed area in Figure 1a–c highlights the UCSC-based BLAT result of P*_VDAC1_*, P*_VDAC2_*, and P*_VDAC3_* matching with functional elements positioned within the region of accessible chromatin that define the nearest active promoter sequence. The structural organization of *VDAC* genes, transcripts, and the surrounding regions were analyzed.

The transcripts coding the three VDACs functional proteins are reported with the codes ENST00000265333.8 for VDAC1, ENST00000543351.5 for VDAC2, ENST00000022615.9 for VDAC3 corresponding respectively to NM_003374.2, NM_001324088.1, and NM_005662.7 in the Refseq database of NCBI.

Several transcript variants from the Ensemble database are also indicated for each *VDAC* isoforms. Most of the splice variants have the same exons composition compared to the coding transcript but differ in the length of the 5′ and 3′. Some of them are processed transcripts, other features retained intron, and for *VDAC3*, two are involved in the nonsense-mediated decay mechanism. It is not known whether the other splice variants identified have any functional biological role. However, gene expression data collected from the NIH Genotype-Tissue Expression project (GTEX), reports their expression, including the non-protein-coding transcripts.

The 600 bp-selected promoter region shows a high degree of overlap with the identification of the CpG island and the RNA polymerase II binding site close to the TSS, also confirmed by ChIP-seq data of chromatin-state model and the enrichment levels of the H3K4me1 and H3K4me3 histone marks, were chosen as the best predictors of transcription and open chromatin elements available among the UCSC regulation tracks.

### 2.2. VDAC Gene Transcription by Expression Atlas Resources

The gene expression profile was highlighted through the analysis of available high-throughput data that have been included in international collaborative projects aiming to characterize human genome expression and regulation. In this work, data revised and curated by the Expression Atlas of EMBL-EBI [32], from GTEx [33] and RIKEN functional annotation of the mammalian genome 5 (FANTOM) project [34], was reported that made interesting comparisons with results obtained with RNA-seq methodology. The expression patterns of the three *VDAC* isoforms depict the level of VDAC transcripts distribution in different human tissues.

**Figure 2:**
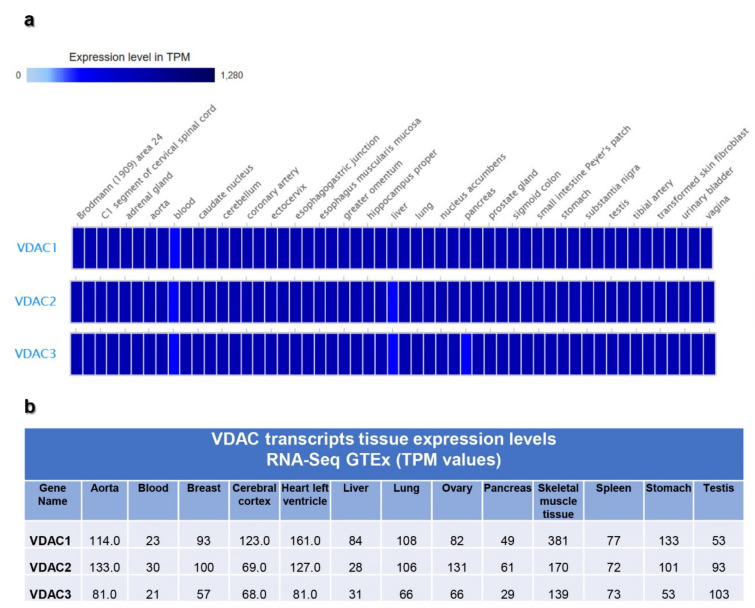
Human *VDAC* genes expression by Genotype-Tissues-Expression (GTEx) database. RNA-seq data from Genotype-Tissues-Expression (GTEx) project were collected from the Expression Atlas repository, where all data are manually curated and subject to standardized analysis pipelines. The data are displayed in a heatmap (**a**) with different colors representing a range of transcripts per kilobase million (TPM) mRNA expression levels. The range of expression levels reported for *VDAC* genes is between 0 and 1280 TPM, as indicated by the bar. In panel (**b**), the specific expression values of each human VDAC isoforms, for representative tissues, are reported.

The RNA-Seq expression data from GTEx obtained using human tissue samples from post-mortem individuals, show that the level of VDAC mRNA expression seems comparable among the three isoforms but with a prevalence of VDAC1 (Figure 2a,b). A relevant result is that all VDAC isoforms are expressed at the highest level in skeletal muscle and heart in comparison to other tissues. VDAC1 and VDAC3 isoforms are expressed with a similar score, while VDAC2 is expressed to a minor extent. Among the other tissues, we noticed that both VDAC1 and VDAC3 are represented in different portions in the brain with a higher score than VDAC2. However, the presence of VDAC1 and VDAC3 in brain regions seems to be differentiated since the former isoform is more expressed in diencephalon while VDAC3 in the telencephalon. A similar situation can be observed in organs forming the digestive apparatus. The specificity of VDAC isoforms expression can be highlighted in other tissues where one isoform is present with a high score, and the other two are not significantly expressed. For example, VDAC1 is more expressed in skin tissues and the kidney. VDAC2 is more specifically expressed in bladder, vagin, cervix/ectocervix. VDAC3 level of expression is particularly higher in testis.

**Figure 3:**
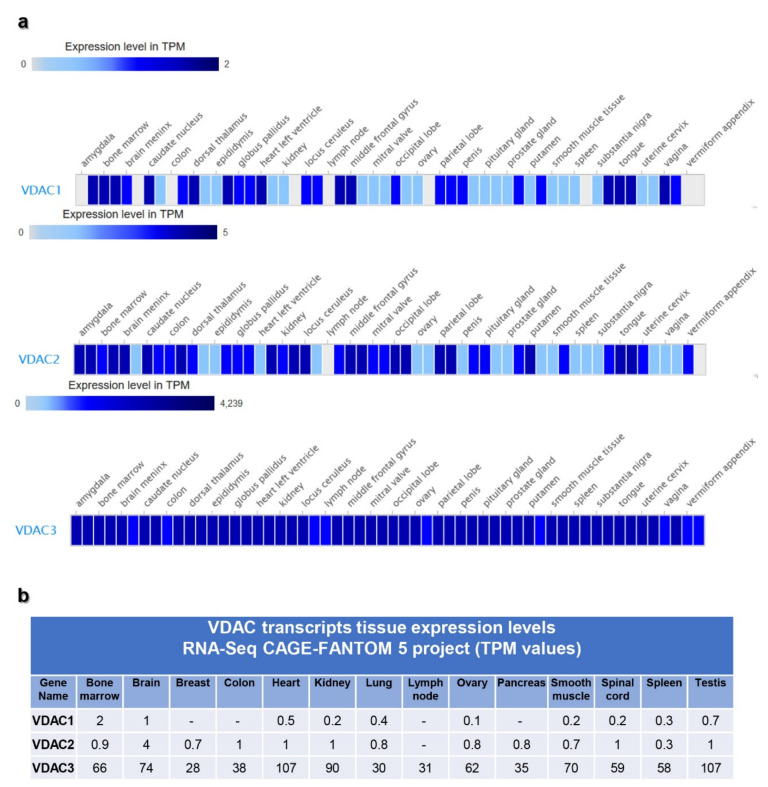
Human *VDAC* genes expression by RIKEN functional annotation of the mammalian genome 5 (RIKEN FANTOM 5) project. RNA-seq cap analysis gene expression (CAGE) data from the RIKEN FANTOM 5 project was collected from the Expression Atlas repository, where all data are manually curated and subject to standardized analysis pipelines. The data are displayed in a heatmap (**a**) with different colors representing a range of TPM mRNA expression levels. In Panel (**b**), the specific expression values of each human VDAC isoform, for representative tissues, are reported.

RNA-seq cap analysis gene expression (CAGE) VDAC transcript expression was selected from the RIKEN FANTOM 5 project. It is well known that the enrichment of mRNA obtained using this technique is a map of TSS and gene promoter activity. In Figure 3, the first relevant result emerging from this analysis was the higher expression level of VDAC3 transcripts when compared to VDAC1 and VDAC2 in all the human tissues tested. Indeed, VDAC3 mRNA expression fell within a higher range of TPM (transcripts per kilobase million) values than that of the other two isoforms. Even if the expression of VDAC1 and VDAC2 transcripts fell in the same range, the latter isoform was the most expressed.

Although the data from the banks are based on experimental results, some comparison between them might look unexpected. The CAGE approach, indeed, is more focused on identifying productive RNA as mRNA and ncRNA. Further work will be necessary to explain in its entirety the regulation of these genes.

In Figure 2, VDACs mRNA levels are reported for some representative tissues. According to the literature and other databases of transcripts expression, VDAC1 and VDAC2 are mainly represented in bone marrow, brain, testis, heart, tongue. However, in the FANTOM 5 project dataset, the VDAC2 level is doubled compared to VDAC1. Although VDAC3 mRNA expression overcame VDAC1 and VDAC2, the tissues with a higher level of its expression were confirmed to be heart, testis, muscles. The data emerging from this analysis highlighted for the first time the prevalence of *VDAC3* gene transcription on other isoforms reflecting a higher promoter activity.

With this analysis, we can also confirm that VDAC isoforms are ubiquitously expressed in tissues, even if with different specificity for each isoform.

### 2.3. VDAC Isoforms Comparative Expression in HeLa Cells

**Figure 4:**
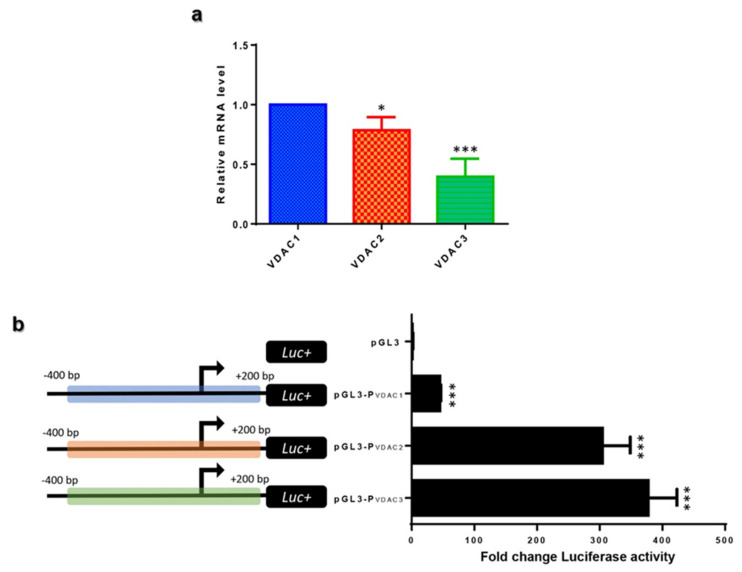
Experimental analysis of human *VDAC* gene expression and their promoter activity in HeLa cells. (**a**) Human *VDAC* genes expression. *VDAC* genes expression was detected by Real-Time PCR as described in methods. After normalization with the housekeeping gene β-actin, the variation of VDAC2 and VDAC3 transcripts was expressed using human VDAC1 as reference. The ΔΔC*t* method was applied. (**b**) VDAC promoter activity detection. To study the promoter activity, 600 bp sequence encompassing the transcription start site (TSS) (from −400 to +200 in the gene sequence) was placed upstream of the luciferase gene in pGL3 plasmid. The assay was performed in HeLa cells transfected with P*_VDAC1_*-pGL3, P*_VDAC2_*-pGL3, P*_VDAC3_*-pGL3 constructs after 48 h of transfection. Luciferase activity of cell lysates was calculated by referring to empty-pGL3 transfected cells and following normalization with Renilla activity. Three independent experiments were performed and results statistically analyzed by one-way ANOVA. A value of *p* < 0.05 was taken as significant. Significance was determined as reported and indicated as *p* < 0.05, * *p* < 0.01, and *** *p* < 0.001.

VDAC isoforms transcription was analyzed in HeLa cells, and a comparison of their expression level was performed. mRNA of the *VDAC1* gene was established as a reference gene, and quantification of VDAC2 and VDAC3 mRNA level was reported relative to VDAC1. In Figure 4a, VDAC2 transcript amount was slightly lower than VDAC1 mRNA showing a value of 0.78, while VDAC3 transcripts were almost half of VDAC1 with a value of 0.39. VDAC3 mRNA was the less expressed transcript among the three isoforms. The data obtained confirm VDAC transcription expression, as revealed by a GTEx data analysis and previous experimental results obtained by us [35].

### 2.4. VDAC Genes Transcriptional Promoter Activity

A 600 bp genomic region encompassing the TSS of *VDAC* genes was identified as a putative promoter named P*_VDAC1_*, P*_VDAC2_*, and P*_VDAC3_* and utilized for experimental characterization. These sequences were cloned in front of the Luciferase (Luc) reporter gene to study the activity of the human *VDAC* gene promoters in HeLa cells. Luciferase activity, driven by the indicated *VDAC* promoters, was compared among the three isoforms, as shown in the histogram of Figure 4b. Surprisingly VDAC1, the most represented isoform, held the less active promoter, which drove the transcriptional activation 10 and 8 folds lower than *VDAC3* and *VDAC2* promoters. These experimental results are consistent with the predominant transcriptional activity of *VDAC3* and *VDAC2* emerging from the FANTOM 5 project, suggesting a mechanism of fine regulation of *VDAC* genes expression. 

### 2.5. Characterization of VDAC Genes Core Promoters

**Figure 5:**
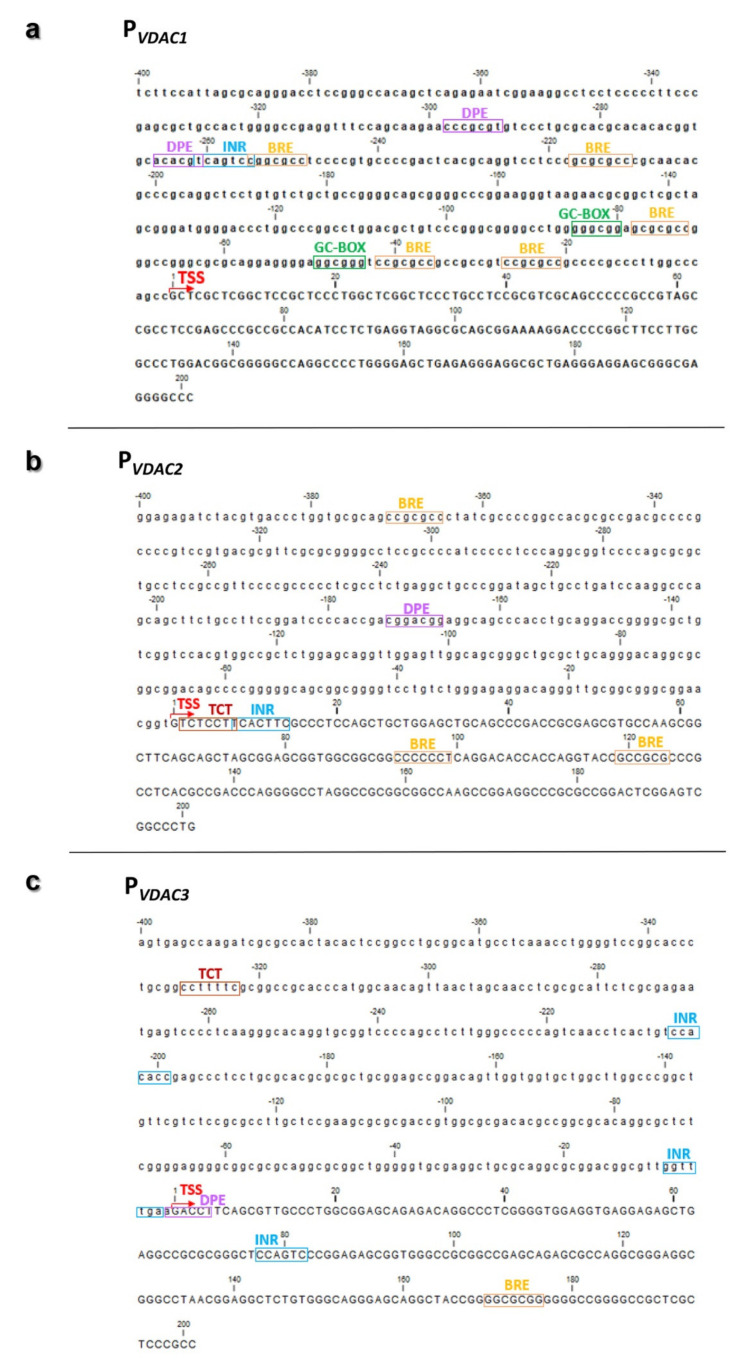
Canonical core promoter elements of human *VDAC* gene isoforms. The results of core promoter elements identified for *hVDAC1*, *hVDAC2*, and *hVDAC3* genes by predictive tools are the sequence stretches with a high scoring consensus based on position weight matrix (PWM). (**a**) P*_VDAC1_* (chr5:133,340,230-133,340,830; hg19) encompassed an Inr element (at −261 bp), two GC-boxes (at −85 bp; −49 bp), five B recognition element (BRE) motifs (at −255 bp; −217 bp; −78 bp; −42 bp; −27 bp), and two downstream promoter element (DPE) motifs (at −298 bp; −266). (**b**) P*_VDAC2_* (chr10:76,970,184-76,970,784; hg19) a Polypyrimidine initiator (TCT) motif, as an alternative Inr (at +2 bp), Inr element (at +8 bp), two BRE motifs (at + 93 bp; +119 bp), a DPE motif (at −173 bp). (**c**) P_VDAC3_ (chr8:42,248,998-42,249,598; hg19) encompasses three Initiator element (Inr) elements (at −205 bp; −8 bp; +77 bp), a TCT (at −329 bp), a DPE (at −1 bp) and a BRE (at +170 bp). TSS site is indicated by a red arrow. Nucleotide sequence before TSS is shown in lowercase.

Very limited information is available regarding the core promoter organization of VDAC genes. Using different predictive strategies through EPD, YAPP Eukaryotic Core Promoter Predictor, and ElemeNT, we built an overview of the most relevant core promoter elements captured with the higher consensus match and functionally recommended scores (*p*-value ≥ 0.001), which we schematically represented for P*_VDAC1_* (Figure 5a), P*_VDAC2_* (Figure 5b), and P*_VDAC3_* (Figure 5c). The promoter region of each *VDAC* gene lacks a canonical TATA box but contains the Initiator element (Inr), downstream promoter element (DPE), and B recognition element (BRE). As typically observed in TATA-less promoters, multiple GC-boxes are required, and Inr and DPE are functionally analogous to the TATA box as they cooperate in the binding of TFIID in the transcription [36]. In P_VDAC2_ (Figure 5a) and P*_VDAC3_* (Figure 5c) a non-canonical initiation site termed the TCT motif (polypyrimidine initiator) was identified. The polypyrimidine stretch proximal to the 5′ end of these genes was a target for translation regulation, oxidative and metabolic stress, or cancer-induced differential translational regulation by the mTOR pathway [37].

### 2.6. Characterization of VDACs’ Transcriptional Regulators

Identifying the upstream regulators of *VDAC* genes will allow a better understanding of the biological role that each isoform plays in the cell. Thus, *VDAC* genes were characterized for the TFBSs by scanning the promoter sequences with three different bioinformatics tools (Genomatix, Jaspar, UniBind), and the results were overlapped to find the most relevant TF families that regulate VDAC gene expression. We used a search window of −400 to +200 bp around the TSS.

**Figure 6:**
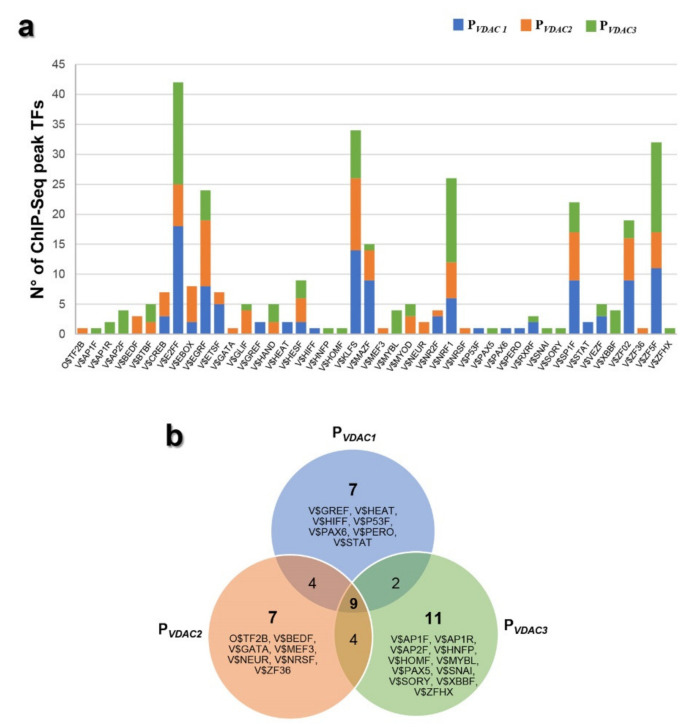
Identification of ChIP-seq peak regions in the human VDAC promoters. (**a**) The histogram shows the number of transcription factor binding sites (TFBS) experimentally validated by ChIP-Seq data (ENCODE project v3) among those predicted by the software Genomatix (MatInspector) in P*_VDAC1_*, P*_VDAC2_*, and P*_VDAC3_* sequences. (**b**) Venn diagram showing the number of common and unique predicted binding sites that overlap with a ChIP-Seq region in P*_VDAC1_*, P*_VDAC2_*, and P*_VDAC3_*, based on Genomatix analysis.

In Figure 6a, the histogram shows every TFBS family found on the *VDAC* promoters’ sequences that were predicted by MatInspector software (Genomatix v3.10) and experimentally validated by ChIP-Seq data (ENCODE project v3). The ChIP-Seq peaks of V$E2FF, V$EGRF, V$KLFS, V$NRF1, V$MAZF, V$SP1F, V$ZF02, V$ZF5F were numerically the most overrepresented as highlighted by the bioinformatic prediction, confirming the importance of these factors in the regulatory network of *VDAC* genes. These binding sites are known to participate in several biological processes, such as cell growth, proliferation and differentiation, development, inflammation and tumorigenesis [38,39,40,41,42,43,44]. Among them, V$NRF1 is the master regulator of genes encoding mitochondrial proteins, and V$E2FF is a family of factors involved in the control of the cell cycle. The results of Figure 6a were reorganized in a Venn chart showing the transcription factors binding sites shared by *VDACs* promoter sequences and those exclusively present in each *VDAC* gene promoter, as reported by Genomatix analysis (Figure 6b).

### 2.7. Analysis of VDACs Common Transcription Factor Binding Sites (TFBSs)

The overlap between Genomatix results and data extracted from JASPAR and UniBind databases highlights the occurrence of four families of shared TFBSs in promoter regions of *VDAC* genes. A comprehensive location of both sets of TFBSs was also extracted from ENCODE ChIP-seq peaks (Table 1).

The detection of common TFBS clusters indicates that these different classes of TFs participate in many similar activities and are mainly involved in cell proliferation and differentiation, apoptosis, and metabolism regulation. Therefore, it is possible to divide different classes of TFs involved in the control of *VDAC* genes into three functional categories: The first is represented by V$E2FF (E2F-myc activator/cell cycle) transcription factors, affecting various processes of cell cycle regulation [45]. The second group includes members of V$EBOX (E-box binding factors) and V$KLFS (Krueppel-like transcription factors) families, which are essential transcription factors that regulate a large number of cellular processes, such as metabolism, cell proliferation, differentiation, apoptosis, and cell transformation [46,47]. The third group comprises V$NRF1 (Nuclear respiratory factor 1) family, closely connected with mitochondrial biogenesis, DNA damage signaling, and tumor metabolism [48,49,50].

### 2.8. Analysis of VDACs Unique Transcription Factor Binding Sites (TFBSs)

The data extracted from Genomatix, Jaspar, and UniBind, were collected to obtain information about families of TFBS exclusively found in each promoter sequence: they thus define the unique TFs controlling each single *VDAC* isoform. We found in the P*_VDAC1_* sequence (Table 2) four unique TFBS families: V$AHRR (AHR-arnt heterodimers and AHR-related factors) which are required, together with HIF-1α factor, for the cell response to hypoxia [51]; V$ETSF (Human and murine ETS1 factors) that includes NRF2, a regulator of mitochondrial biogenesis and redox homeostasis [52]; V$HEAT (heat shock factors) a family of proteins crucial for cell stress response [53]; V$PBXC (PBX-MEIS complexes), also known as pre-B cell leukemia family, that includes regulators of cell development, survival, invasion, and proliferation [54].

As concerning the P*_VDAC2_* sequence (Table 3), we identified several binding sites recognized by regulators known to be associated with the nervous system development and general core promoter elements. Among them: V$BEDF (BED subclass of zinc-finger proteins) includes ZBED, which controls cell growth and differentiation in cone photoreceptors and Müller cells of the human retina [55]; V$BRAC (Brachyury gene, mesoderm developmental factor), is involved in the commitment of T helper (Th) cells [56]; V$CLOX (CLOX and CLOX homology (CDP) factors), a crucial regulator of the neuronal differentiation in the brain [57]; V$MEF3 (MEF3 binding sites) a family that includes regulators of skeletal muscle development [58]; V$NEUR (NeuroD, Beta2, HLH domain), comprising the basic helix–loop–helix factors Ascl1 and OLIG2 involved in neural development and differentiation [59]; TF2B (RNA polymerase II transcription factor II B) a core promoter element [60]; V$ZFXY (Zfx and Zfy—transcription factors), a family of transcription factors implicated in mammalian sex determination [61].

The results of P*_VDAC3_* analysis (Table 4) showed a distribution of binding sites for TFs involved in the control of various cellular processes including cell differentiation, proliferation, apoptosis, and gametogenesis: V$BCL6 (BED subclass of zinc-finger proteins), a critical regulator of B cell differentiation [62]; V$CDXF (Vertebrate caudal related homeodomain protein) involved in development and maintenance of trophectoderm [63]; V$FOX (Forkhead (FKH)/Forkhead box (Fox)), including important regulators of development, organogenesis, metabolism, and cell homeostasis [64]; V$SOHLH (Spermatogenesis and oogenesis basic helix–loop–helix) transcription regulators of male and female germline differentiation [65]; V$HMG (High-Mobility Group family), including factors that regulate neuronal differentiation and also play important roles in tumorigenesis [66]; V$HOMF (Homeodomain transcription factors) involved in central nervous development [67]; V$IRFF (Interferon regulatory factors) required for differentiation of hematopoietic cells [68]; V$LBXF (Ladybird homeobox (lbx) gene family) that plays a critical role in embryonic neurogenesis and myogenesis and in muscle mass determination [69]; V$MYBL (cellular and viral myb-like transcriptional regulators) that controls cell cycle progression, survival, and differentiation [70]; V$SMAD (Vertebrate SMAD family of transcription factors) that includes factors responsible for several cellular processes, including proliferation, differentiation, apoptosis, migration, as well as cancer initiation and progression [71]; V$XBBF (X-box binding factors) family involved in the control of development and maintenance of the endoplasmic reticulum (ER) in multiple secretory cell lineages [72].

**Figure 7:**
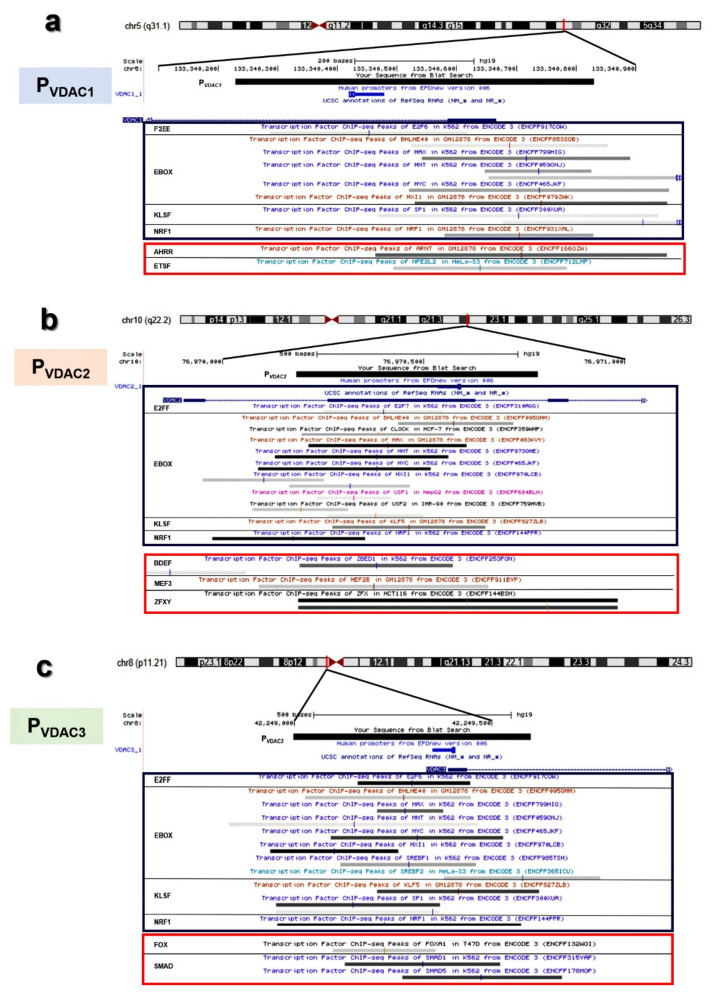
Identification of common and unique transcription factor binding site clusters of *VDACs* promoter sequences. Distribution of a common set (enclosed in a blue box) and specific sets (enclosed in a red box) of Transcription Factor Binding Sites (TFBSs) in *VDAC* isoforms promoters as reported in different cell lines by ChIP-Seq analysis in the ENCODE Project (shown as vertical color bars in the gray segments). (**a**) *hVDAC1*, (**b**) *hVDAC2*, and (**c**) *hVDAC3*.

In Figure 7a–c, a magnification of *VDAC* promoters analyzed from the UCSC Genome Browser is overlapped with experimental data proving the transcriptional activity of this genomic region. Based on Genomatix results on distinct and shared TFBSs at promoter regions of P*_VDAC1_*, P*_VDAC2_*, and P*_VDAC3_*, a comprehensive location of both sets of TFBSs was extracted from ENCODE ChIP-seq peaks. These findings are also supported by TFBS enrichment analyses from JASPAR and UniBind database. In the graphical view, the most interesting peaks of TFBS found were located in overlapping positions of the promoter for different cell lines. Moreover, these validated TFBSs fell in the genomic region corresponding to *VDAC* promoters studied in this work.

The determination of common TFBSs appears to corroborate shared biological properties, as well as a high degree of functional conservation and cooperation among the three isoforms, while the mapping of unique TFBSs robustly supported by different databases suggests a different biological role.

## 3. Discussion

To understand the specialized biological role of VDAC isoforms, simultaneously expressed in cells, we performed a characterization of VDAC transcripts expression and promoters’ structure and function. To have a general but reliable picture of *VDAC* genes structure, expression, and regulation, we undertook a study of VDAC isoforms in the main available public resource reporting high throughput data of international collaborative projects.

### 3.1. Structure of VDAC Genes, Transcripts, and Promoters

First of all, a general view of *VDAC* genes, transcript variants, and promoter regions feature by in silico analysis through the UCSC genome browser was reported. For each *VDAC* gene, several different transcript splice variants were identified: they do not vary in the coding region but mainly in their 5’-UTR and 3’-UTR length. Other variants were processed transcripts. Others present retained intron, and for VDAC3, two were involved in nonsense-mediated decay mechanism. The variability of UTR sequences let us hypothesize differentiated mechanisms of transcript regulation and expression context for each variant. The 3′-UTR sequence may be a target of translation regulation by miRNA or interference. Many publications indeed reported the identification of miRNA molecules targeting all three VDACs transcripts but, in particular, VDAC1 [73]. The 5′-UTR region variability might be associated with alternative promoter usage and activation in a different expression context. However, no information is available on the human *VDAC* promoters and/or other regulative regions to explain transcripts expression.

### 3.2. VDAC Expression in Expression Atlas Repository

For this reason, we wanted to focus our study on the characterization of the main promoter driving each *VDAC* gene transcript expression. First of all, we selected from the Expression Atlas repository, the data derived from the RNA-seq CAGE RIKEN FANTOM 5′ project and the RNA-seq GTEx projects.

Generally, although the expression profile of VDACs transcription presents a differentiated level in different tissue or cell types, all three isoforms were ubiquitously expressed [2]. The level of VDAC1 and VDAC2 transcripts was comparable, while VDAC3 was found less expressed [35]. Surprisingly, analyzing the data set of the FANTOM 5 project we found that the number of transcripts from VDAC3 expression overcame VDAC2, but, in particular, VDAC1 whose transcripts resulted in being scarcely represented in all tissues, compared to the other VDACs. The special version of RNA-seq methodology based on cap analysis of gene expression adopted by the FANTOM5 consortium allowed the identification of active TSS located on the 5′-end of transcribed mRNA, which was not necessarily associated to the protein-coding transcript. Based on this evidence, we selected the main promoter region found in the Eukaryotic Promoter Database (EPD) associated with the main protein-coding transcripts, and we confirmed by luciferase reporter assay that the *VDAC3* promoter had the highest transcriptional activity and the *VDAC1* promoter was, on the contrary, the least active. The molecular mechanisms related to the difference in *VDAC* genes expression and regulation are still to be investigated. The finding that we highlighted here might be associated with the biological role of VDAC proteins. *VDAC* genes are housekeeping genes; thus a basal expression level is expected in any tissue. From the scarce information available in the literature, it was hypothesized that *VDAC* genes are subject to quantitative regulation of expression. We found, in a previous work, that NRF-1 and HIF can modulate the activity of *VDAC1* promoter [74]: thus, the introduction of other TF or stimuli can change the expression pattern of the gene.

Moreover, the higher promoter activity of *VDAC3* might also be interpreted as being potentiality maintained by the cells to respond to a particular and still unknown stimulation through VDAC3 increased expression in specific conditions promptly. Another possibility is that VDAC1 transcripts are more stable than VDAC3 ones, thus explaining the need for a higher production of VDAC3 mRNAs to get the right translation levels.

Thus, some results might look unexpected without a deeper analysis of every factor affecting the activity of the promoter. Further work urges to explain in its entire complexity the regulation of these genes.

### 3.3. VDAC Genes Core Promoters

With the aim to explore the mechanism of *VDAC* genes transcription regulation, we started a systematic analysis of human *VDAC* promoters to highlight their structural and functional features. *VDAC* gene core promoter organization is similar to most of TATA-less human core promoters of ubiquitously expressed genes. Abundant GC regions, alternative binding sites Inr, DPE, and BRE for the basal transcription factors take over the function of the TATA box sequence. Moreover, VDACs genes, as most human protein-coding genes are lacking the TATA-box, are characterized by a long 5′-UTR region suggesting the presence of alternative TSS employed for the expression of distinct products in different contexts or tissues. As reported in the *VDAC* gene structure organization, the occurrence of several transcripts could explain their expression associated with different conditions.

### 3.4. Transcription Factors Binding Sites Common to Any VDAC Gene

We also characterized the main transcription factors regulating the activity of *VDAC* promoter regions, looking for the transcription factors binding sites (TFBSs). The information we gained by bioinformatic analysis suggested the central role of VDAC protein expression in regulating mitochondrial function in fundamental cell processes. We recognized TFBS shared by the three *VDAC* promoters, as well as single promoters’ unique sites. Among the common ones, the majority of identified TFs classes belonged to the E2F-myc activator/cell cycle (E2FF), Nuclear respiratory factor 1 (NRF1), SP1, Krueppel-like transcription factors (KLFS), E-box binding factors (EBOX) families, which participate in many similar activities but are prevalently involved in cell proliferation and differentiation, apoptosis, and metabolism regulation [38,39,40,41,42,43,44]. TFBS for E2FF and NRF1 transcription factor family members were also numerically the most represented in all the three *VDAC* promoters. *VDAC* promoters were mainly characterized by a large number of recognition sites for E2FF and NRF1 transcription factors, which were found associated by chromatin immunoprecipitation with microarrays (ChIP-on-chip) to a significant subset of genes implicated in mitochondrial biogenesis and metabolism, other than mitosis, cytokinesis, cell cycle control, grow, proliferation [75]. Many identified TFBS were located in the proximal core promoter acting as co-regulators for general transcription factor activation and chromatin regulation. In particular, some of them, SP1, KLFS, EBOX, NRF1 rich in GC content, have an important role in epigenetic control of promoter, suggesting a more complex regulation of this gene [76].

### 3.5. Transcription Factors Binding Sites Specific to Each VDAC Gene

Search for unique transcription factor binding sites in the promoters of *VDAC* isoforms allowed us to hypothesize their involvement in specialized biological functions. However, the transcription factors specific for each *VDAC* genes were always correlated to essential functions ensuring cell survival and functionality. In general, these processes require a noteworthy energy cost: metabolism maintenance, development, organogenesis, dysfunction of mitochondria in pathology are some examples.

The families of transcription regulators identified as unique in *VDAC1* promoter suggests that this isoform was probably selected by evolutionary process to have the prevalent role of channel protein in the mitochondrial outer membrane in physiological context and, in particular, when altered conditions force the cells to restore the mitochondria energetic balance [74]. These observations are corroborated by several experimental evidences showing the involvement of VDAC1 in regulating many cellular and mitochondrial events in pathology or stress conditions through the interaction with specific protein [77].

VDAC2 was indicated as the isoform carrying out channel function and governing apoptosis and autophagy in various contexts [5]. Analysis of *VDAC2* promoter highlighted the presence of different factors specially involved in developing specialized tissues and the organogenesis process as unique among *VDAC* promoters. Most of these factors are related to nervous system genesis and development.

*VDAC3* is controlled by the most active promoter: it is particularly rich in GC repetitions, suggesting an epigenetic control mechanism able to reduce the expression of transcripts. Factors binding sites found in *VDAC3* promoter belong to various families, but those involved in the development of germinal tissues, organogenesis, and sex determination are the most abundant. In addition, in this case, the experimental evidence reported in the literature confirms the crucial role of VDAC3 in fertility [13].

## 4. Materials and Methods

### 4.1. Bioinformatic Analysis of Promoter Region

Human promoter retrieval for *hVDAC1* (NM_003374), *hVDAC2* (NM_001324088), and *hVDAC3* (NM_005662) genes was carried out by high-quality promoter resource EPDnew version 006 (https://epd.epfl.ch). For study purposes, P*_VDAC1_* (chr5:133,340,230-133,340,830; hg19), P*_VDAC2_* (chr10:76,970,184-76,970,784; hg19), and P*_VDAC3_* (chr8:42,248,998-42,249,598; hg19) were the promoter sequences extended from −400 bp to +200 bp relative to annotated Transcription Start Site (TSS) of basal EPD promoter sequences (VDAC1_1; VDAC2_1; VDAC3_1). Tools used to scan for canonical core promoter elements and synergistic combinations were EPD Promoter Elements Page, YAPP Eukaryotic Core Promoter Predictor (http://www.bioinformatics.org/yapp/cgi-bin/yapp_intro.cgi), and ElemeNT (http://lifefaculty.biu.ac.il/gershon-tamar/index.php/resources). The core promoter elements with the higher consensus match and functionally recommended scores (*p*-value ≥ 0.001) were selected.

Analysis of transcription factor binding site (TFBS) clusters was done by Genomatix software suite (Genomatix v3.10). MatInspector application was used to identify potential binding sites for transcription factors (TFBSs) in input sequence using the Matrix Family Library version 11.0 for core promoter elements in vertebrates with a fixed matrix similarity threshold of 0.85 [78], and the ChIP-Seq regions of all transcription factors assigned to the matrix family were checked with ENCODE data from UCSC Genome Browser (TFBS clusters (V3) from ENCODE data).

TFBSs enrichment analysis was also performed using JASPAR (JASPAR CORE vertebrates collection 2020), the largest open-access database of position-specific scoring matrices derived from experimentally validated TFBS [79], by selecting the predicted binding sites with a *p*-value ≥ 0.001, and UniBind, a comprehensive map of direct TF–DNA in the human genome based on public ChIP-seq datasets [80].

In view of implementing the TFBS clusters analysis, all information relevant to the genomic structure and DNA regulatory elements related to three *VDAC* isoforms were investigated using the UCSC Genome Browser (https://genome.ucsc.edu). DNA regulation tracks of the UCSC Genome Browser and some information on promoter sequence and TFBS predictions are currently available on GRCh37/hg19.

### 4.2. Gene Expression Data Retrieval

Gene expression data were collected from the Expression Atlas of EMBL-EBI open science resource (https://www.ebi.ac.uk/gxa/home), a publicly available repository of selected RNA-seq, microarray, and proteomics datasets manually curated and analyzed through standardized analysis pipelines [32]. The Baseline Atlas database containing the RNA-seq experiments regarding the expression of genes in tissues under physiological conditions was consulted, and data from the Genotype-Tissue-Expression (GTEx) [33] and Functional Annotation of the Mammalian Genome 5 (FANTOM) project [34] were selected for VDAC isoform expression analysis. GTEx database contains data from quantitative measurements of transcripts based on RNA-seq of tissues. Instead, data reported in the FANTOM database was obtain by an alternative approach to RNA-seq, named cap analysis gene expression (CAGE), based on the sequencing of the 5′-end of capped mRNA molecules. The data are displayed in a heatmap with different colors representing a range of TPM mRNA expression levels. Generally, the level is represented by grey (expression level is below cutoff. 0.5 TPM or FPKM); light blue (expression level is low, between 0.5 and 10 TPM or FPKM); medium blue (expression level is medium, between 11 and 1000 TPM or FPKM ); dark blue (expression level is high, more than 1000 TPM or FPKM ); white box (no data available). Transcript expression levels of the three *VDACs* genes were selected for the most common tissues and represented by a histogram and reported in a table.

### 4.3. Quantitative Real-Time PCR

HeLa cells were plated in 25 cm^2^ flasks at a rate of 0.6 × 10^6^. After 48 h of incubation, total RNA was extracted using the ReliaPrep RNA cell mini-prep system (Promega, Madison, WI, USA), according to the manufacturer’s instructions. RNA concentration and purity were measured by a spectrophotometer, and 2 µg was used to synthesize cDNA by a QuantiTect Reverse Transcription kit (Qiagen, Hilden, Germany). Real-time amplification was performed in a Mastercycler EP Realplex (Eppendorf) in 96-well plates. The reaction mixture contained 1.5 µL cDNA, 0.2 µM gene-specific primers pairs (hVDAC1, hVDAC2, hVDAC3, β-actin), and 12.5 µL of master mix (QuantiFast SYBR Green PCR kit, Qiagen). Three independent experiments of quantitative real-time were performed in triplicate for each sample. Analysis of relative expression level was performed by the ΔΔC*t* method using the housekeeping *β-actin* gene as an internal calibrator and *VDAC1* gene as reference.

### 4.4. Plasmid Constructs

A putative promoter region of 600 bp encompassing the TSS of the human *VDACs* genes was selected from GenBank and cloned into pGL3 basic vector (Promega) for transcriptional activity study. The construct P*_VDAC1_* contained the sequence (chr5:133,340,230-133,340,830; hg19) derived from *VDAC1* NM_003374, P*_VDAC2_* contained the genomic trait (chr10:76,970,184-76,970,784; hg19) from *VDAC2* NM_001324088 and for P*_VDAC3_*, the sequence was (chr8:42,248,998-42,249,598; hg19) of *VDAC3* NM_005662.

### 4.5. Promoter Reporter Assay

HeLa cells were plated at a density of 0.3 × 10^6^ cells/well in a 6-well plates. After 24 h, cells were transfected with 800 ng of pGL3 constructs and 20 ng of pRL-TK renilla reporter vector by Transfast transfection reagent according to the manufacturer’s protocol (Promega), and after 48 h, cells were lysed. Luciferase activity of cell lysate transfected with pGL3 promoter constructs was detected with the Dual Luciferase Assay (Promega) according to the manufacturer’s instructions. The activity of firefly luciferase relative to renilla luciferase was expressed in relative luminescence units (RLU). variation of luminescence units of treated samples relative to control, were indicated as fold increase (FI).

### 4.6. Statistical Analysis

Data are presented as means ± SD of results obtained from three independent experiments. All experiments were performed with n = 3 biological replicates and n = 3 technical replicates. Statistical significance was determined by one-way ANOVA. Significance was determined as reported and indicated as * *p* < 0.05, ** *p* < 0.01, and *** *p* < 0.001.

## 5. Conclusions

In the present work, we proposed a general overview of the structural and functional organization of VDAC isoform promoters, cross-referencing public available data sources, bioinformatics prediction, and experimental data. The purpose of this work was, thus, to present experimental data extrapolated from Databases, with our experimental confirmation of the promoter sequences and activity by artificial constructs. From this analysis, emerges the essential function of the family of VDAC proteins in the regulation of energetic mitochondrial metabolism in physiological and pathological cell life. Moreover, we shed some new light on the molecular mechanisms that explain the differences among the three VDAC isoforms. It is becoming increasingly clear that the most known specialized functions of each VDAC isoforms are connected with the organization of the “button room” that decides the transcriptional activity of their genes and were produced by evolution. With this work, we established a starting point to pave the way for a deeper and wider analysis, an aim that we are pursuing.

## Figures and Tables

**Table 1:** Voltage-dependent anion-selective channels (VDACs) common Transcription Factor Binding Sites (TFBSs). The sign ✓ indicates the presence of the TFBS in the promoter selected by a specific database.

		Rate of Frequency in Database								
Matrix Family	Description	P*_VDAC1_*			P*_VDAC2_*			P*_VDAC3_*		
		*Genomatix*	*Jaspar*	*Unibind*	*Genomatix*	*Jaspar*	*Unibind*	*Genomatix*	*Jaspar*	*Unibind*
V$E2FF	E2F-myc cell cycle regulator	✓	✓	✓	✓	✓	✓	✓	✓	-
V$EBOX	E-box binding factors	✓	✓	✓	✓	-	-	✓	✓	✓
V$KLFS	Krueppel like transcription factors	✓	✓	✓	✓	✓	✓	✓	✓	✓
V$NRF1	Nuclear respiratory factor 1	✓	✓	✓	✓	✓	✓	✓	✓	✓

**Table 2:** VDAC1 unique Transcription Factor Binding Sites(TFBSs). The sign ✓ indicates the presence of the TFBS in P*_VDAC1_* sequence selected by a specific database.

		Rate of Frequency in Database		
Matrix Family	Description	P*_VDAC1_*		
		*Genomatix*	*Jaspar*	*UniBind*
V$AHRR	AHR-arnt heterodimers and AHR-related factors	-	-	✓
V$ETSF	Human and murine ETS1 factors	✓	-	-
V$HEAT	Heat shock factors	✓	-	-
V$PBXC	PBX - MEIS complexes	-	-	✓

**Table 3:** VDAC2 unique Transcription Factor Binding Sites (TFBSs). The sign ✓ indicates the presence of the TFBS in P_VDAC2_ sequence selected by a specific database.

		Rate of Frequency in Database		
Matrix Family	Description	P*_VDAC2_*		
		*Genomatix*	*Jaspar*	*UniBind*
V$BEDF	BED subclass of zinc-finger proteins	✓	-	-
V$BRAC	Brachyury gene, mesoderm developmental factor	-	-	✓
V$CLOX	CLOX and CLOX homology (CDP) factors	-	-	✓
V$MEF3	MEF3 binding sites	✓	-	-
V$NEUR	NeuroD, Beta2, HLH domain	✓	-	-
O$TF2B	RNA polymerase II transcription factor II B	✓	-	-
V$ZFXY	Zfx and Zfy—transcription factors	-	-	✓

**Table 4:** VDAC3 unique Transcription Factor Binding Sites (TFBSs). The sign ✓ indicates the presence of the TFBS in P*_VDAC3_* sequence selected by a specific database.

		Rate of Frequency in Database		
Matrix Family	Description	P*_VDAC3_*		
		*Genomatix*	*Jaspar*	*UniBind*
V$BCL6	POZ domain zinc finger expressed in B-Cells	-	-	✓
V$CDXF	Vertebrate caudal related homeodomain protein	-	-	✓
V$FOX	Forkhead (FKH)/Forkhead box (Fox)	-	-	✓
V$SOHLH	Spermatogenesis and oogenesis basic helix-loop-helix	-	✓	-
V$HMG	HMG family	✓	-	-
V$HOMF	Homeodomain transcription factors	✓	✓	-
V$IRFF	Interferon regulatory factors	-	-	✓
V$LBXF	Ladybird homeobox (lbx) gene family	-	✓	-
V$MYBL	Cellular and viral myb-like transcriptional regulators	-	✓	-
V$SMAD	Vertebrate SMAD family of transcription factors	-	✓	-
V$XBBF	X-box binding factors	✓	-	-

## Abbreviations

AHRR	AHR-arnt heterodimers and AHR-related factors
BCL6	B-cell lymphoma 6 protein
BEDF	BED subclass of zinc-finger proteins
BRAC	Brachyury gene, mesoderm developmental factor
BRE	B recognition element
CAGE	Cap analysis gene expression
CDXF	Vertebrate caudal related homeodomain protein
CLOX	CLOX and CLOX homology (CDP) factors
DB	Database
DPE	Downstream promoter element
E2FF	E2F-myc activator/cell cycle
EBOX	E-box binding factors
ER	Endoplasmic Reticulum
ETSF	Human and murine ETS1 factors
FANTOM	Functional ANnotation of the Mammalian Genome
FPKM	Fragments Per Kilobase Million
FOX	Forkhead (FKH)/ Forkhead box (Fox)
GTEX	Genotype-Tissue Expression
HEAT	Heat shock factors
HMG	High-Mobility Group family
HOMF	Homeodomain transcription factors
Inr	Initiator Element
IRFF	Interferon regulatory factors
KLFS	Krueppel-like transcription factors
LBXF	Ladybird homeobox (lbx) gene family
MEF3	MEF3 binding sites
MYBL	Cellular and viral myb-like transcriptional regulators
NEUR	NeuroD, Beta2, HLH domain
NRF1	Nuclear respiratory factor 1
PBXC	PBX-MEIS complexes
SMAD	Vertebrate SMAD family of transcription factors
SOHLH	Spermatogenesis and oogenesis basic helix-loop-helix
TF2B	RNA polymerase II transcription factor II B
TFBSs	Transcription Factor Binding Sites
TPM	Transcripts Per kilobase Million
TSS	Transcription Start Site
VDAC	Voltage-Dependent Anion selective Channel
XBBF	X-box binding factors
ZFXY	Zfx and Zfy-transcription factors
